# P-283. Evaluating Missed Opportunities for HIV Diagnosis in a Statewide Health System

**DOI:** 10.1093/ofid/ofaf695.504

**Published:** 2026-01-11

**Authors:** Timothy C Adkins, Nicole Bryan, Jesse M Thompson

**Affiliations:** West Virginia University, Danville, WV; West Virginia University, Danville, WV; West Virginia University, Danville, WV

## Abstract

**Background:**

HIV is an ongoing public health burden which can be controlled with interventions such as ART. In recent years, outbreaks of HIV infection have occurred related to the opioid epidemic. Inconsistencies in screening high risk patients in rural communities at health care encounters may lead to delays in care, more opportunistic infections, and further HIV transmission. Addressing screening disparities in rural communities can improve patient outcomes and reduce future outbreaks. WVU Medicine is the largest health system in WV and includes 25 hospitals located throughout the state. This study retrospectively examines 101 recently diagnosed HIV patients who received care at WVU Medicine for patterns in missed opportunities for early HIV diagnosis.

**Table 1:**
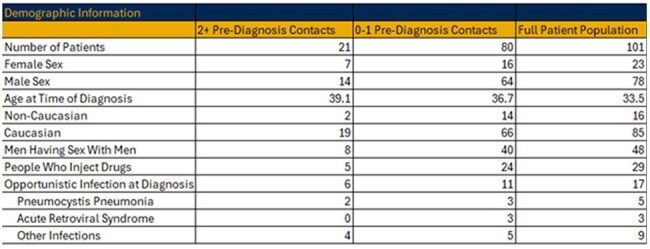
Demographic Information, by Factor

**Table 2:**
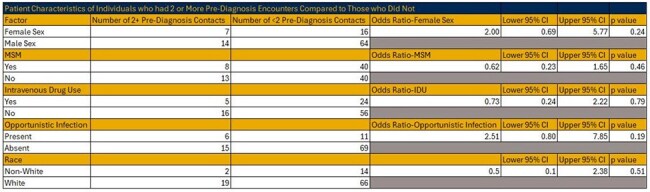
Patient Characteristics of Individuals who had 2 or More Pre-Diagnosis Encounters Compared to Those who Did Not

**Methods:**

The electronic health records of 101 patients with newly diagnosed HIV who established care with the Ryan White funded WVU Positive Health Clinic from 1/1/2020-1/1/2025 were reviewed. The number of inpatient and outpatient encounters that occurred within the WVU Medicine Healthcare System one year prior to HIV diagnosis or in the period since last HIV test, were counted. Visits where the patient left prior to testing or treatment, or refused testing, were not counted. The presence of characteristic opportunistic infections was also tallied. Statistical analysis for description of binary associations was performed with the calculation of odds ratios and associated confidence intervals.

**Figure 1:**
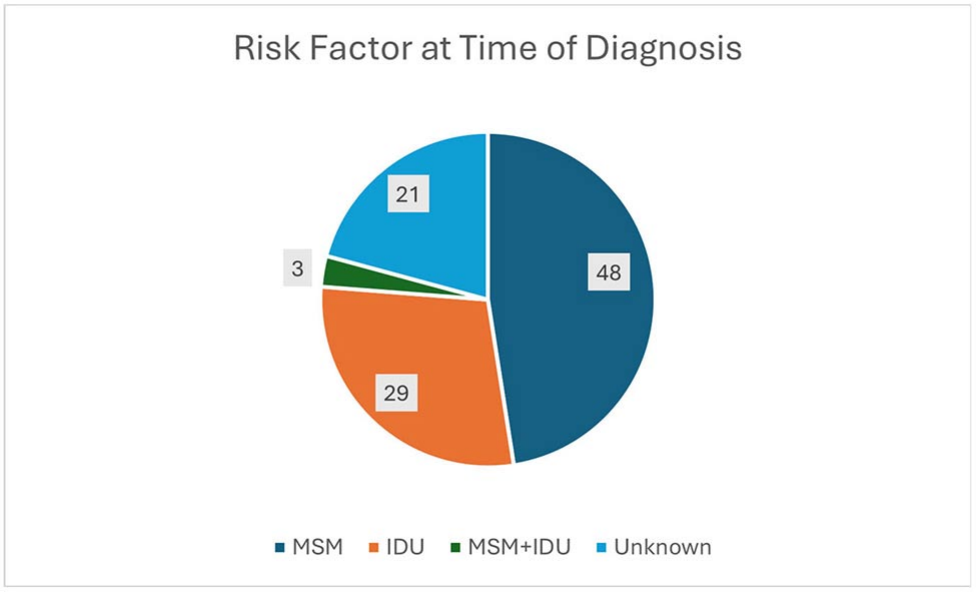
Risk Factor at Time of Diagnosis

**Figure 2:**
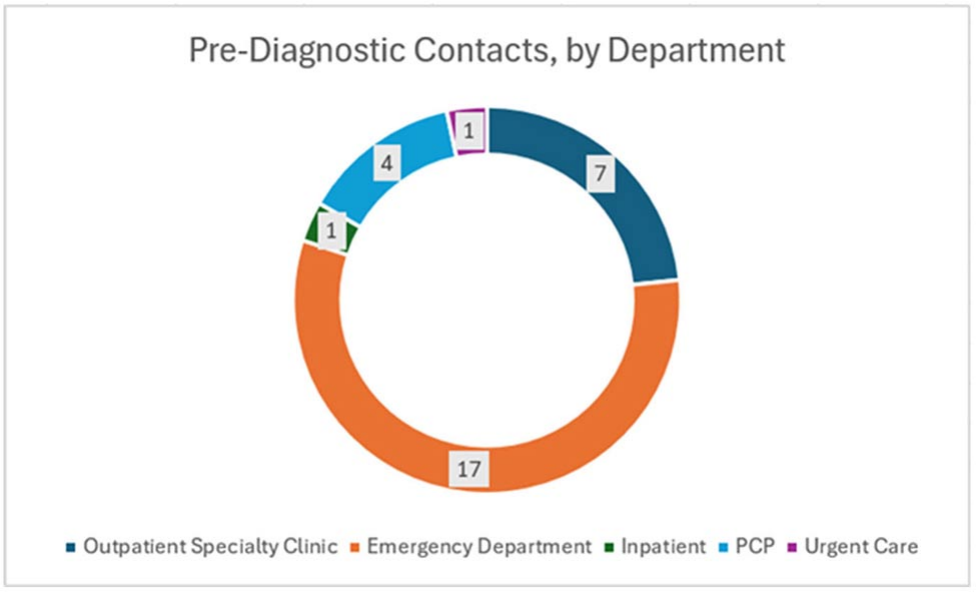
Pre-Diagnostic Contacts, by Department

**Results:**

21 of the 101 patients in the study had two or more encounters with the health care system prior to diagnosis. No identified risk factor- MSM, IDU, or opportunistic infection- had a statistically significant impact on whether there were two or more healthcare contacts prior to diagnosis. The most common setting for these was the Emergency Department.

**Conclusion:**

Missed testing opportunities in the Emergency Department are a consequence of volume and triage of priorities. No single risk factor had a statistically significant effect on the number of healthcare encounters prior to diagnosis, though due to size the statistical power of the study is small. Improving identification and screening procedures for patients at community and rural access hospitals, especially in Emergency Departments, may reduce the number of missed new HIV diagnoses.

**Disclosures:**

All Authors: No reported disclosures

